# The “Opercular” Approach to Orbital Reconstruction after Orbital Exenteration Following Rhino-Orbital-Cerebral Mucormycosis: A Novel Method

**DOI:** 10.1055/s-0044-1793947

**Published:** 2024-12-04

**Authors:** Bharatendu Swain, A. Shalini Sampreethi, Shravya C.

**Affiliations:** 1 Department of Plastic and Reconstructive Surgery, Aakar Asha Hospital, Hyderabad, Telangana, India; 2 Department of Oral and Maxillofacial Surgery, Aakar Asha Hospital, Hyderabad, Telangana, India

**Keywords:** mucormycosis, rhino-orbital cutaneous mucormycosis, ROCM, orbital exenteration, orbital reconstruction, opercular approach

## Abstract

**Background** Rhino-orbital cutaneous mucormycosis (ROCM) leading to orbital exenteration can be debilitating functionally as well as psychosocially. Orbital reconstruction following exenteration for mucormycosis has centered on volume filling with local, regional, or free flaps. This case series is built on the original idea of a bilayered operculum at the orbital inlet.

**Materials and Methods** The opercular approach comprises an inner layer of hinged orbital mucosa and an outer layer of nasofacial flap in most cases, or alternatives, for lining or cover.

**Results** Eight cases of ROCM treated using the opercular approach are presented with satisfactory results and minimal complication. Four of the eight patients treated by this method remained free of complications after 1year.

**Conclusion** This technique is simple, does not require microsurgical expertise, and is less time-consuming and less expensive.

## Introduction

Rhino-orbital cutaneous mucormycosis (ROCM) is an opportunistic infection that occurs in immunosuppressed patients. ROCM can result in high mortality or dysfunction and disfigurement. The COVID-19 wave saw a large number of patients of ROCM who underwent orbital exenteration. 1

The conventional approach to reconstruction has been filling of the orbital volume defect by various methods including local, regional, and free flaps. 2 Elements of reconstruction included obliteration of the orbital mucosa, which is often unstable, with ulcers, and filling of the orbital volume defect with soft tissue and a transverse oval of skin representing the eyelids. The other goal in reconstruction has been readying the orbit to receive a bone anchored eye prosthesis. 3

As an original idea, the senior author has adopted the “opercular” approach in orbital reconstruction, which closes off the orbital volume defect externally and still retains the ability to accommodate a future prosthesis. The residual volume defect becomes part of a larger rhino-antro-orbital space and drains into the nasal or oral cavity. Treated patients have expressed satisfaction with the procedure and chosen not to opt for an ocular prosthesis—bone integrated or external. 4

## Materials and Methods

Fourteen patients with exenterated orbits (13 with type IIIc and 1 with type IVa ROCM 5 ) and associated palatal defects, partial maxillectomy or septal defects, were referred to our institution between July 2022 and January 2024.

Eight of 14 patients with orbital exenteration due to ROCM, without palatal defect (in 2 cases, the palatal defect was repaired at an earlier stage), local disease free, and medically fit to undergo surgery, who underwent reconstruction of the orbital defect via the opercular approach, were included in the study. Patients with existing palatal defects were excluded from the study.

Eight of the 14 patients underwent reconstruction of the exenterated orbit via the opercular approach.

Each patient was taken up for surgery with a clear disease-free interval of 6 months or more and in cases of unstable orbital mucosa (ulcerated or crusted), frequent saline dressings were done for at least 15 days or till complete mucosal healing.

**Fig. 1:**
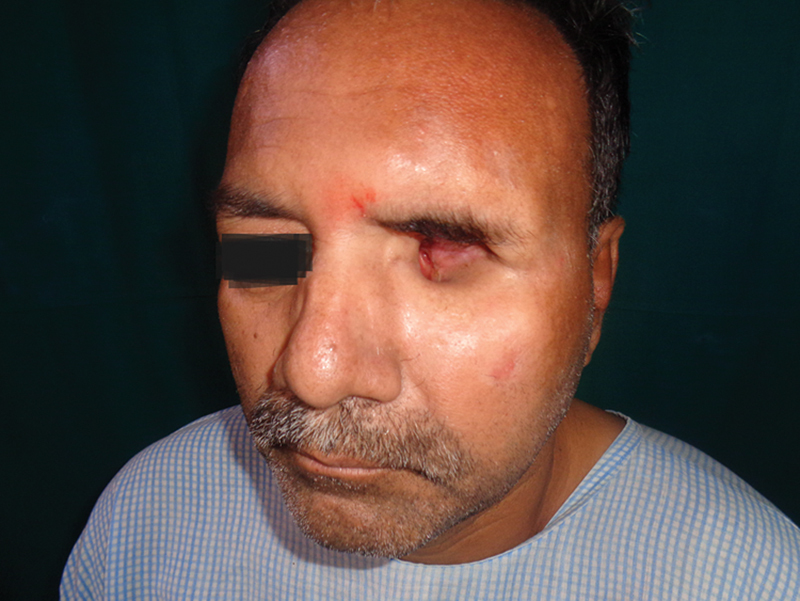
Case 1: Preoperative.

An orbital inlet perimeter incision was made and hinge flaps developed, based posteriorly, closed with inverting mattress sutures of 3–0 polyglactin (Vicryl, Ethicon India), to form an inner layer. In seven of eight cases, the ipsilateral nasofacial flap was advanced to close the external defect, with 4–0 polyamide (Ethilon, Ethicon India), with a suction or corrugated drain kept between the two layers ( Figs. 1 2 3 4 5 6 7 ). In one case with unstable orbital mucosa and sequestrated infraorbital rim, after sequestrectomy, the median forehead flap was used to form the lining instead of the hinge flaps (( Figs. 8 9 10 11 12 ).

**Fig. 2:**
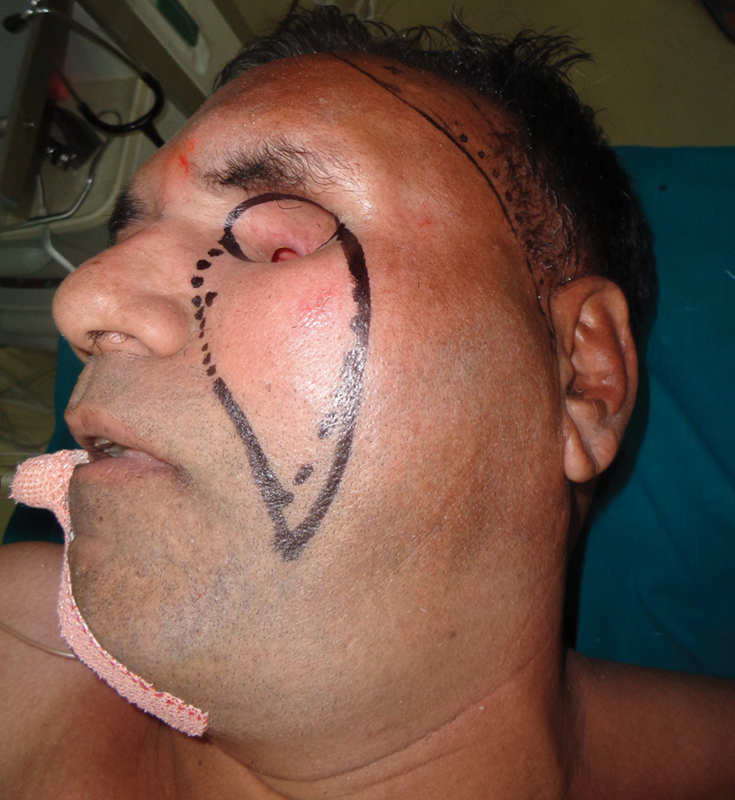
Case 1: Preoperative marking.

**Fig. 3:**
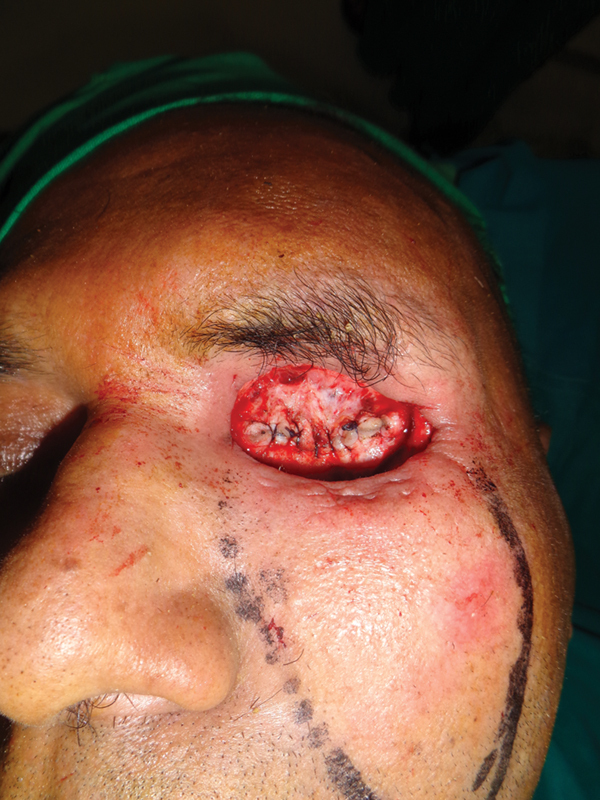
Case 1: Intraoperative view of the inner layer.

**Fig. 4:**
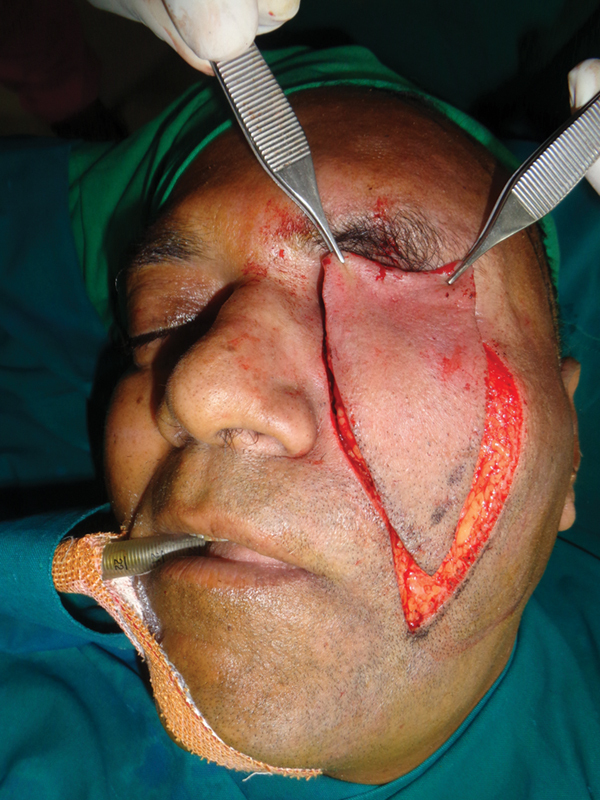
Case 1: Intraoperative view of the nasofacial flap advanced.

**Fig. 5:**
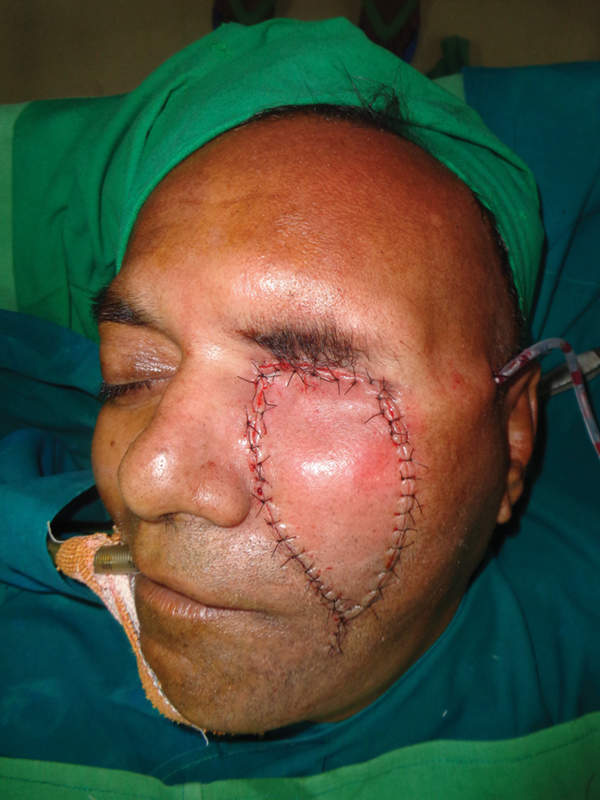
Case 1: Intraoperative view after closure.

**Fig. 6:**
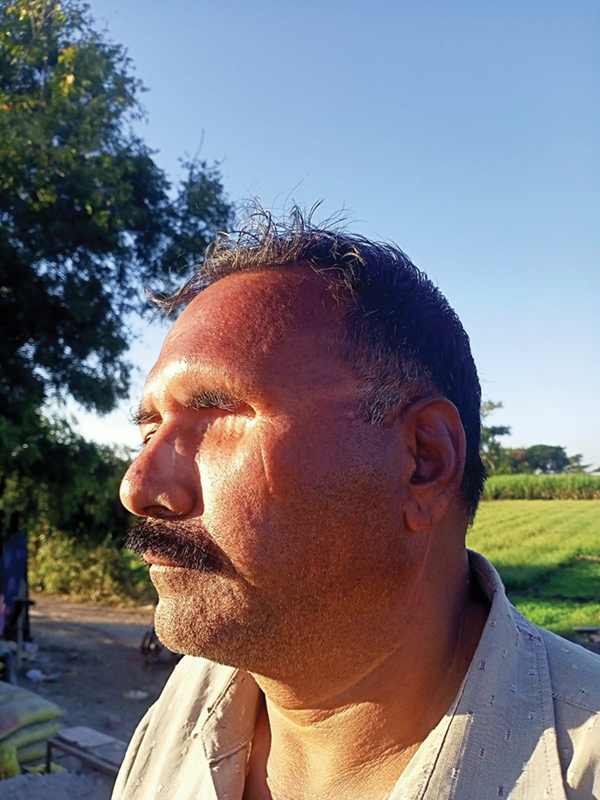
Case 1: Review after 1 year and 5 months. Oblique view.

**Fig. 7:**
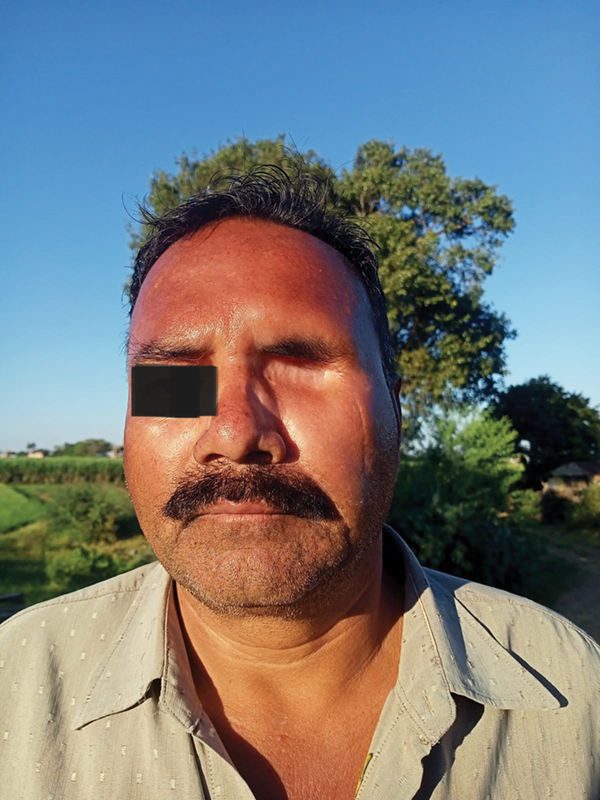
Case 1: Review after 1 year and 5 mo. Frontal view.

**Fig. 8:**
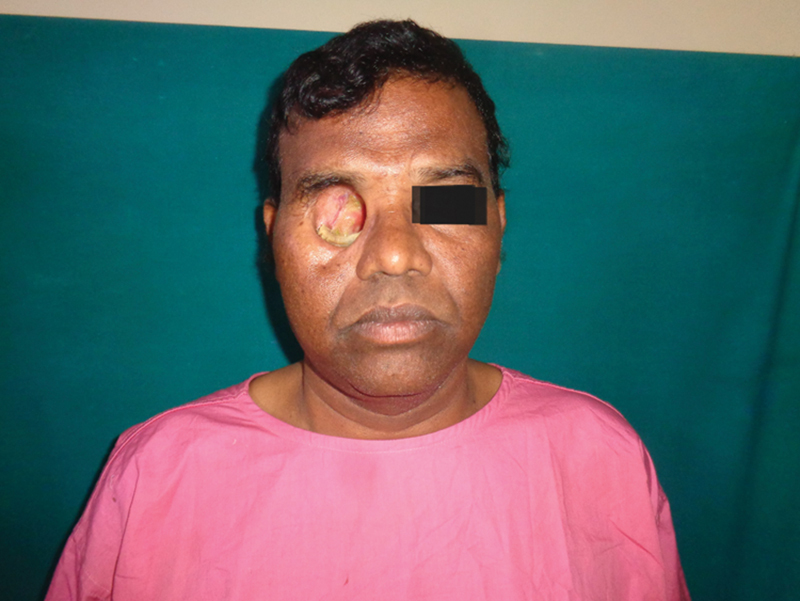
Case 2: Preoperative frontal view.

**Fig. 9:**
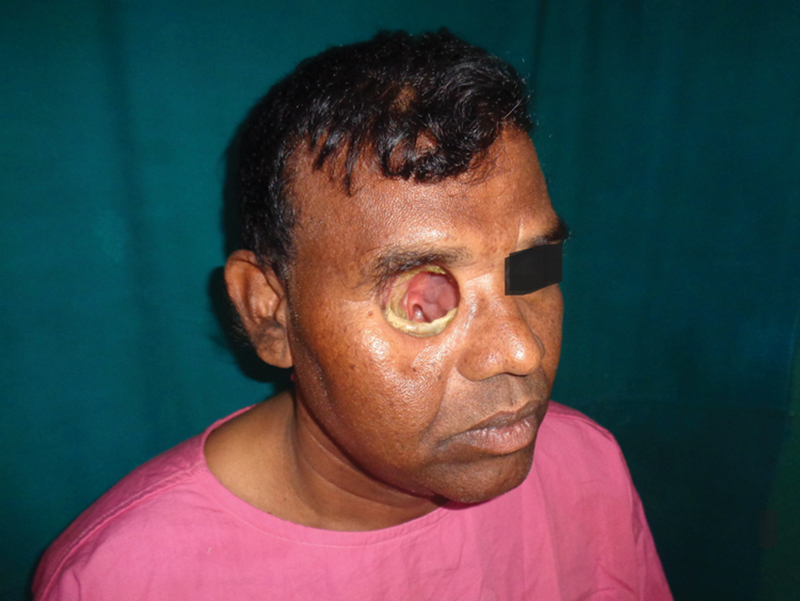
Case 2: Preoperative oblique view.

**Fig. 10:**
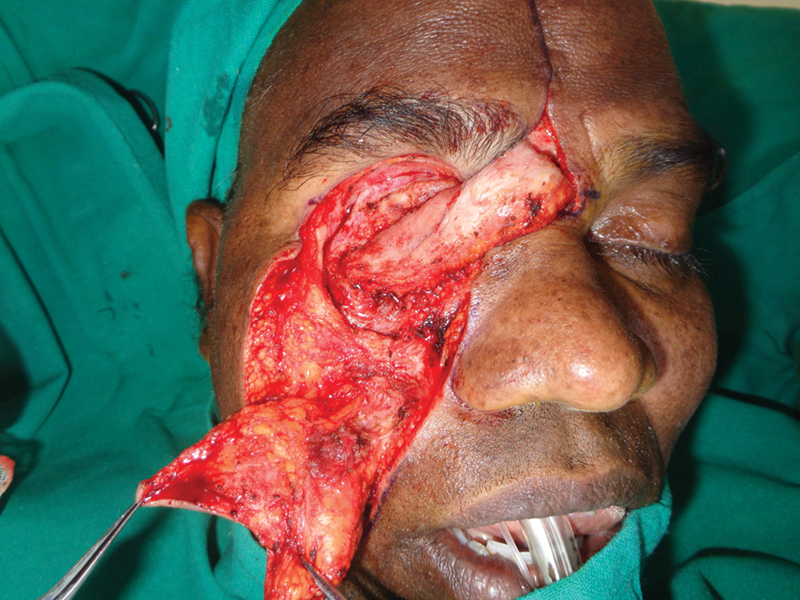
Case 2: Intraoperative view showing the forehead flap for lining and nasofacial flap as cover.

**Fig. 11:**
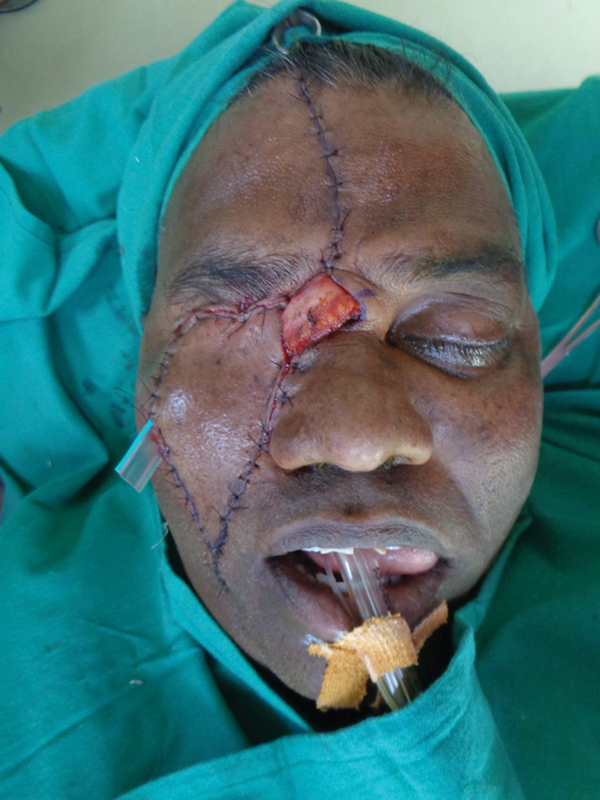
Case 2: Intraoperative view after closure.

**Fig. 12:**
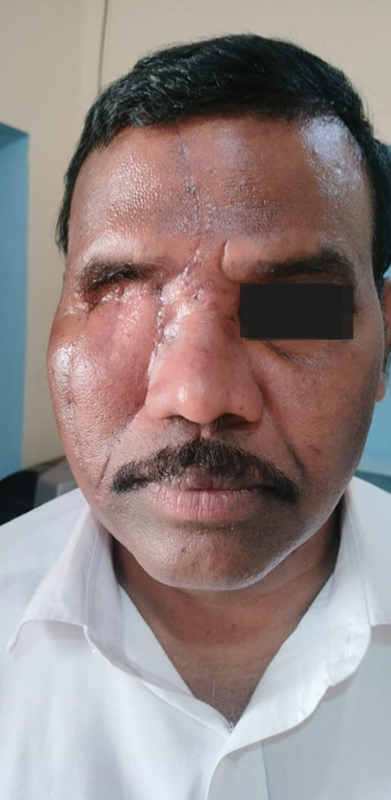
Case 2: Review. Short term.

The suction or corrugated drain was removed after 5 to 6 days and sutures were removed after the 10th day.

**Fig. 13:**
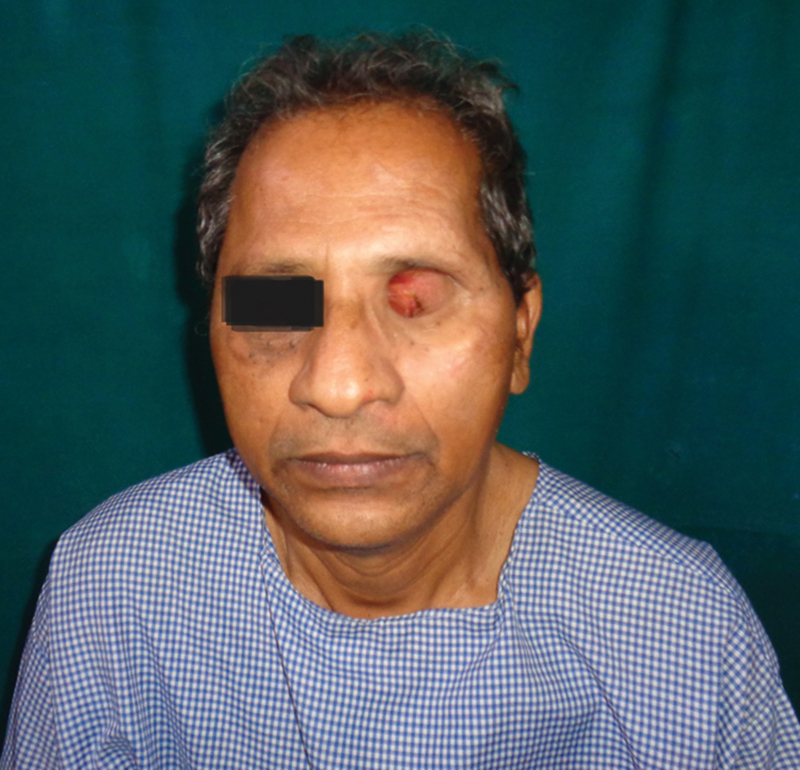
Case 3: Intraoperative view of the inner layer.

All patients were on follow-up for at least 6 months and, where indicated, nasoendoscopy was done to view the inner layer and examine the ipsilateral naso-antral-orbital space for crusting or ulceration. Early crusts that developed in two cases were removed endoscopically; further follow-up endoscopy was done after 10 days' interval to monitor healing ( Figs. 13 14 15 16 17 18 ).

**Fig. 14:**
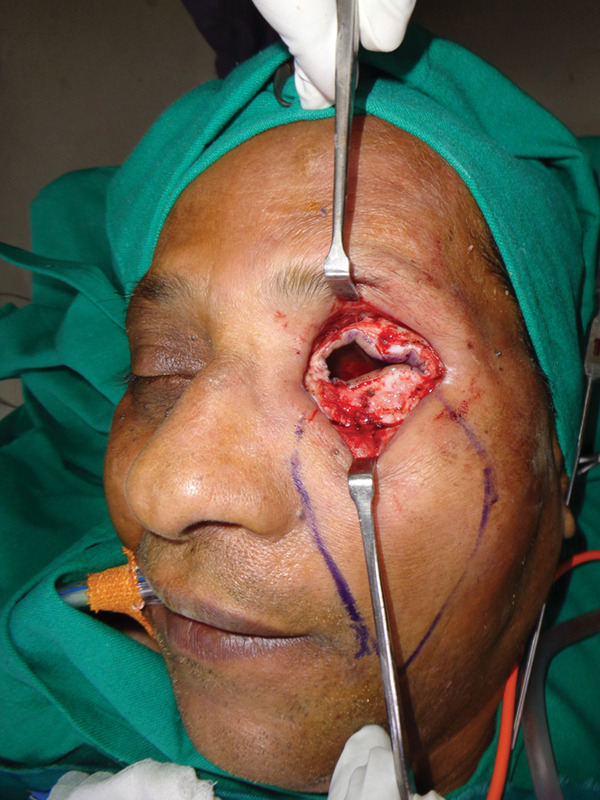
Case 3: Preoperative.

**Fig. 15:**
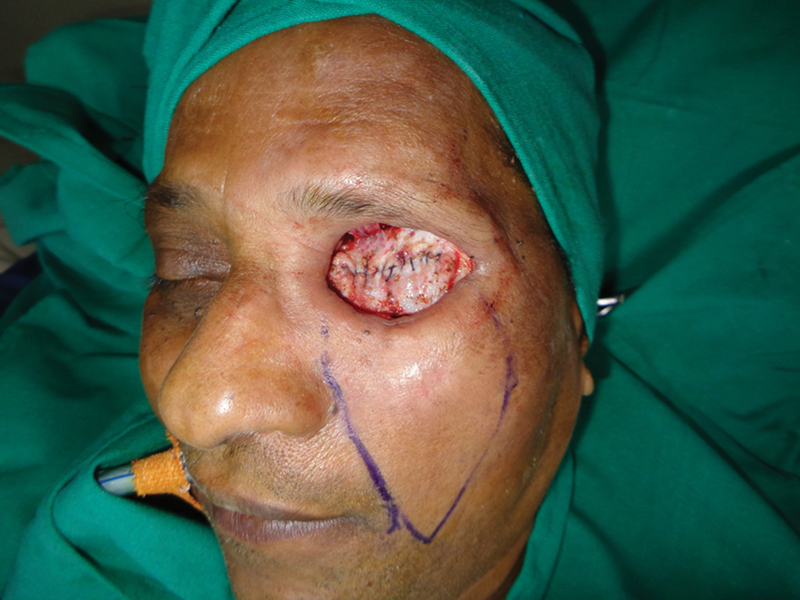
Case 3: Intraoperative view of the hinge flap.

**Fig. 16:**
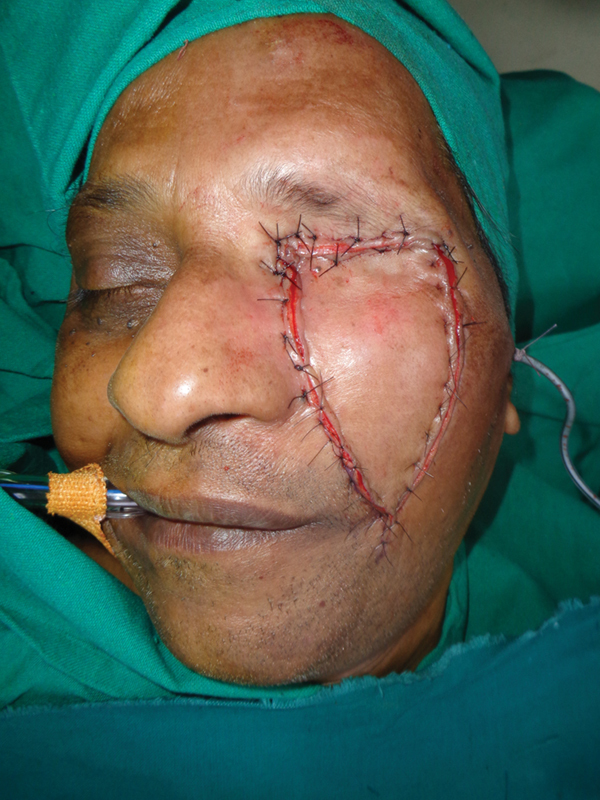
Case 3: Intraoperative view of the nasofacial flap advanced.

**Fig. 17:**
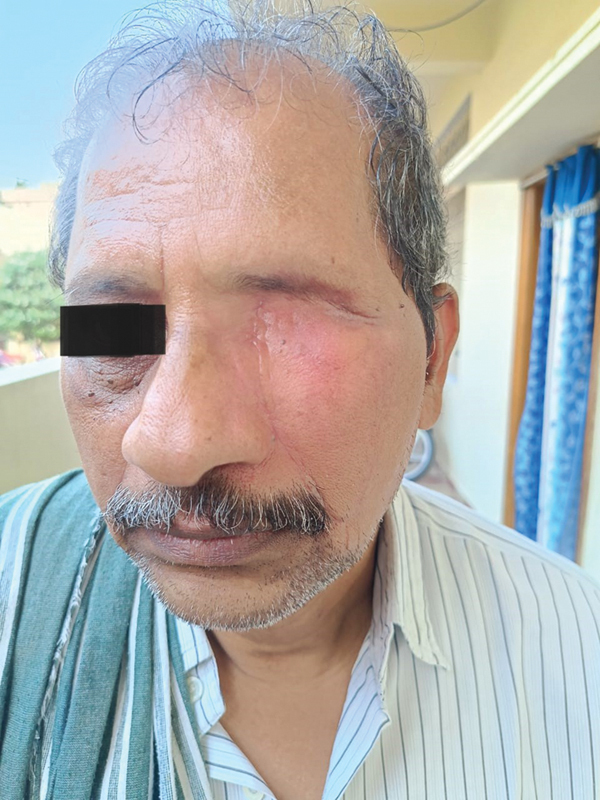
Case 3: Review.

**Fig. 18:**
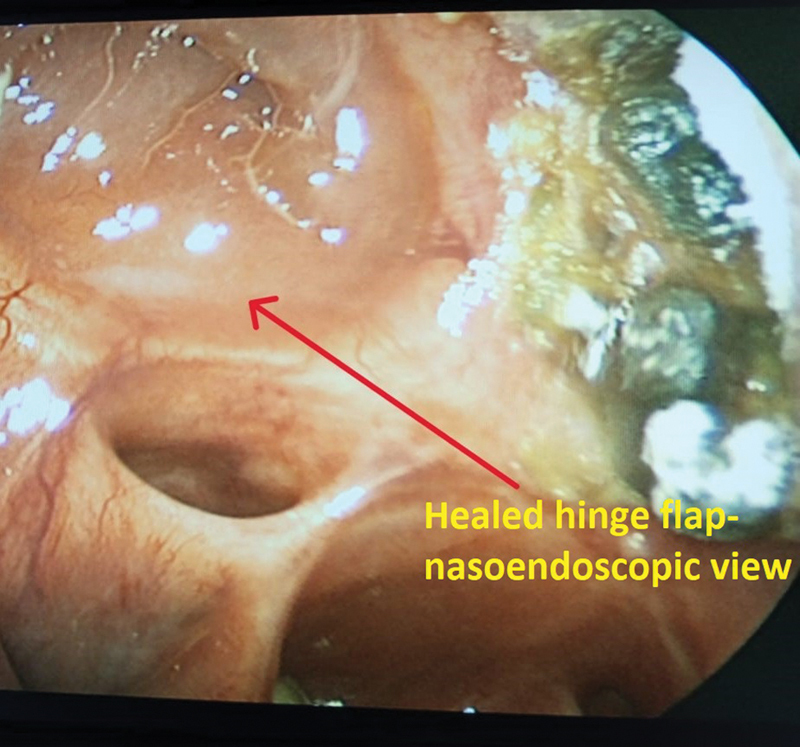
Case 3: Appearance of the hinge flap endoscopically.

At follow-up, all patients were asked to rate their overall satisfaction of facial appearance on a visual analog scale of 1 to 10 and score the sensory apperception of the treated area in comparison to the opposite side on a scale of 1 to 5.

## Results

The eight patients who underwent orbital exenteration for ROCM developed during Covid 2021 were aged 47.2 years on an average (25–58 years; 7 of the 8 patients were in their 50s), predominantly male (7 males and 1 female), with diabetes mellites type II in six of eight patients, hypertension in three patients, and history of transient hemiplegia (magnetic resonance imaging showed thrombosis of intracranial segment of internal carotid artery on left side) in one patient. The average disease-free interval was 8.8 months ( Table 1 ). Four patients required saline dressings for at least 2 weeks where the orbital mucosa was deemed unstable.

**Table 1:** Patient particulars

Sl. no.	Age/sex	Cause of mucormycosis	Comorbidity	Date of orbital exenteration	Duration since cessation of antifungal treatment
1	55/M	COVID: 2021	T2 DM, HTN	June 2021	6 mo
2	37/M	COVID: 2021	T2 DM, HTN	May 2021	8 mo
3	50/M	COVID: 2021	T2 DM	May 2021	9 mo
4	50/M	COVID: 2021	Hemiplegia, T2 DM	June 2021	9 mo
5	58/M	COVID: 2021	HTN	May 2021	12 mo
s6	52/M	COVID: 2021	T2 DM, HTN, >creatinine levels	June 2021	8 mo
7	51/M	COVID: 2021	T2 DM, HTN	May 2021	1 y
8	25/F	COVID: May 2021		May 2021	2 y and 3 mo

Abbreviations: DM, diabetes mellitus; HTN, hypertension.

The seven patients who underwent nasofacial flap cover needed an average surgical time of 2 hours and 8 minutes (including anesthesia) compared with the single case with radial forearm flap cover, which need 6 hours ( Table 2 ).

**Table 2:** Reconstructive surgery details

Sl. no.	Preoperative saline dressings	Lining	Cover	Duration of the surgery	Complications/undesirable outcomes	Further orbital prosthesis	Follow-up	Patient satisfaction	Nerve sensation
		Hinge flap/others	Nasolabial flap/radial forearm flap						
1	None	Yes	NL	135 min	Minor wound dehiscence	No	2 y	8	5
2	Saline dressings (15D)		RFF	6 h	None	No	2 y	8	1
3	Saline dressings (1M)	Yes	NL	150 min	None	No	1 y and 10 mo	10	5
4	None	Yes	NL	50 min	None	No	1 y and 7 mo	10	5
5	Saline dressings (1 M)	Yes	NL	135 min	None	No	6 mo	9	5
6	None	Median forehead flap	NL	180 min	None	No	3 mo	8.5	5
7	Saline dressings (2M)	Yes	NL	130 min	None	No	3 mo	9	5
8	None	Yes	NL	120 min	None		2 mo	8	5

Abbreviations: D, days; M, months; NL, Naso-labial flap; RFF, radial forearm flap.

All patients healed well, except one patient who had a minor wound dehiscence, which was repaired and went on to heal uneventfully. In one patient with a significant medial orbital wall defect and partial maxillectomy, nasoendoscopic removal of crusts was done once, postoperatively, which went on to mucosalize well ( Table 2 ).

The four patients who expressed satisfaction with their appearance at long-term review (>1 year) did not have any specific complaints with regard to the residual orbital volume defect and did not opt for ocular prosthesis at the time of review. The remaining four of the 8 patients expressed satisfaction at this point in time and will be reviewed for at least 1 year. All patients felt relieved at not having to cover the hollow orbits with dressings.

The overall patient satisfaction on a visual analog score after surgery range between 8 and 10.

All patients rated their facial sensation as 5/5, except the one patient who had radial forearm flap cover who scored 1/5 ( Table 2 )

## Discussion

The COVID-19 pandemic affected the entire world on an unprecedented scale. In India, the impact of the second wave in 2021 was huge, with 19.29 million confirmed cases and 242,211 confirmed deaths reported between March 1 and June 30, 2021. 6 Particularly during the second wave, a dangerous complication followed in the form of COVID-19-associated mucormycosis. 7 The Indian Union Health Minister stated that 28,252 cases of mucormycosis were reported from 28 states/union territories in the country, of which 86% cases had a history of COVID-19 infection and 62.3% had a history of diabetes. 8 In total, 2,826 cases of ROCM were reported in a multicenter study 5 in India, of which 78% were diabetics.

After the initial medical management and radical debridement, there was a cohort of cases that had a gaping orbital defect sometimes associated with a maxillary, palatal, or septal defect. The disfigurement, regurgitation of fluids, and crusting led the patients to seek a surgical solution.

The conventional approach in type III ROCM cases involves filling of the orbital volume defect with local flaps, using the temporalis muscle with a skin graft, or using the lateral arm, radial forearm, and anterolateral thigh flap as free tissue transfer. 4 5 Local skin flaps like the forehead flap and cheek rotation flap have been used along with the temporalis muscle 9 ; the defacement, scarring, and hollowing make it an undesirable procedure. The temporalis muscle flap used along with skin graft can have limitations in reaching the medial aspect of the orbital cavity and is used for separating intracranial communication with the orbit. 10 Radial forearm, lateral arm, and anterolateral thigh flaps used as free tissue transfers have the advantage of acting as fillers as well as covering adjacent cheek defects; their use is more common in orbital or cheek malignancy. 11

In our series of 8 of 14 cases of ROCM, which were reconstructed, we developed the original idea of closure of the orbital aperture as an operculum, using the orbital mucosa as a hinge flap to form the inner lining and nasofacial skin as cover in 7 cases; in 1 case, a free radial forearm flap was used as cover and in another the forehead flap was used as lining. This opercular approach simplified the entire procedure of orbital reconstruction, making it quicker and much less expensive and reducing the chances of flap failure. The treated area had a natural “feel” in the cases done with a nasofacial flap cover. Our series demonstrates that filling the orbital volume gap may not be necessary.

On review, patients were quite satisfied with their appearance and the social inclusion gained as they did not have to wear an eye patch all the time. None of the reviewed patients wished to have an ocular prosthesis. All patients achieved social inclusion after the procedure including a temple priest and a church priest, both of whom have gone back to performing their priestly duties.

The drawback of the study is that this approach may not be feasible in cases of extensive periorbital scarring or presence of an oroantral fistula.

This study is limited in terms of persons treated and is an ongoing evolution of technique. However, the ease of execution, simplicity, and cost-effectiveness of the opercular approach make it a desirable option.
